# Heparin binding protein in patients with acute respiratory failure treated with granulocyte colony-stimulating factor (filgrastim) – a prospective, placebo-controlled, double-blind study

**DOI:** 10.1186/1471-2334-13-51

**Published:** 2013-01-30

**Authors:** Kirsi-Maija Kaukonen, Heiko Herwald, Lennart Lindbom, Ville Pettila

**Affiliations:** 1 Intensive Care Unit, Helsinki University Central Hospital, Helsinki, Finland; 2 ANZIC Research Centre, Department of Epidemiology and Preventative Medicine, School of Public Health & Preventive Medicine, Monash University, Melbourne, Australia; 3 Department of Clinical Sciences, Division of Infection Medicine, Lund University, Lund, Sweden; 4 Department of Physiology and Pharmacology, Karolinska Institutet, Stockholm, Sweden; 5 Department of Clinical Sciences, University of Helsinki, Helsinki, Finland

**Keywords:** Filgrastim, G-CSF, Heparin-binding protein, Critically ill, Acute respiratory failure

## Abstract

**Background:**

Heparin Binding Protein (HBP) is released to blood circulation from activated neutrophils in bacterial infections. It is a potential inducer of vascular leakage and precludes the development of septic shock. Filgrastim induces the production of new neutrophils and modulates their bacterial-killing activity. We evaluated the effect of filgrastim on HBP –concentrations in critically ill patients with acute respiratory failure.

**Methods:**

59 critically ill patients with acute respiratory failure were included in this randomised, double-blind, placebo-controlled study of filgrastim 300 micrograms/day or corresponding placebo for 7 days. Plasma samples were drawn on baseline, day 4 and day 7. HBP –concentrations, absolute leukocyte and neutrophil counts were measured.

**Results:**

The median [IQR] HBP concentrations were 23.6 ng/ml [13.9-43.0 ng/ml], 25.1 ng/ml [17.7-35.5 ng/ml] and 15.9 ng/ml [12.6-20.7 ng/ml] in patients receiving filgrastim on baseline, day 4 and day 7, respectively. The HBP concentrations in placebo group were 21.6 ng/ml [16.9-28.7 ng/ml], 13.9 ng/ml [12.0-19.5 ng/ml] and 17.8 ng/ml [13.6-20.9 ng/ml]. At day 4, the filgrastim group had significantly higher HBP –concentrations when compared to placebo group (p < 0.05). No correlation between HBP –concentrations and absolute neutrophil count or P/F –ratios was found.

**Conclusions:**

Filgrastim treatment is associated with increased circulating HBP levels compared to placebo, but the absolute neutrophil count or the degree of oxygenation failure did not correlate with the observed plasma HBP –concentrations.

**Trial registration:**

Clinicaltrials.gov NCT01713309

## Background

Plasma leakage from the vasculature is an important step in the development of septic shock in patients with infections [1]. Heparin binding protein (HBP; also known as azurocidin and CAP37) is an immunomodulatory mediator released from activated neutrophils [2]. The release of HBP is stimulated e.g. when neutrophils adhere to the endothelial lining or when these cells encounter bacterial products in the circulation. The released HBP initiates rearrangement of the endothelial cell cytoskeleton, leading to openings in the endothelial barrier and increased macromolecular leakage to interstitial space [3]. This is an important mechanism of uncontrollable leakage in inflammatory conditions.

In capillary leakage such as septic shock, burns or erysipelas caused by group A Streptococci, elevated levels of HBP have been documented [4-8]. The levels of HBP were elevated up to 12 h prior to the clinical manifestation of septic shock [5].

Pulmonary infections and septic infections are responsible for more than a third of acute respiratory failure leading to ICU admission [9]. In bacterial infections, neutrophils become activated leading to the release of HBP [10]. Filgrastim is a granulocyte colony stimulating factor (G-CSF) which stimulates neutrophil production in bone marrow as well as strengthens their functions, such as phagocytosis and bacterial killing [11]. Increased HBP –levels have been documented in several different bacterial infections [4-6,12]. However, no clinical trial has studied the effect of G-CSF on HBP –levels.

We hypothesized that increased number of circulating neutrophils in response to G-CSF treatment would be associated with increased HBP concentrations in plasma compared to placebo. Accordingly, we analyzed the HBP concentrations in critically ill patients with acute respiratory failure who participated in a prospective, randomised, double-blind study of G-CSF vs. placebo.

## Methods

This is a study of plasma HBP –levels of a previously published trial of G-CSF in critically ill patients [13]. The study was approved by the Ethics Committee of Department of Anaesthesiology in Helsinki University Central Hospital. The notice to the Finnish National Agency for Medicines was submitted 60 days before the initiation of the study as required by Finnish legislation. Informed consent was obtained from the study patients or from a close relative.

### Study patients

During a 16 –month period from February 1997 to June 1998 altogether 636 patients were admitted to the mixed 10 –bed ICU. 59 consecutive patients were included into the study. The inclusion criteria were 1. age >18 years, 2. admission <12 h before study inclusion, 3. intubation <48 h of study inclusion, 4. expected ICU stay >48 h, 5. Informed consent signed. The exclusion criteria were 1. age <18 years, 2. admission >12 h before study inclusion 3. Intubated >48 h before study inclusion, 4. expected stay in ICU <48 h, 5. no informed consent, 6. pregnant or nursing, 7. administration of filgrastim, sargramostin or other biological response modifiers within 7 days, 8. participation in another medicinal trial.

### Study design

The study was a prospective, randomised, double-blind, placebo-controlled trial of filgrastim in patients with acute respiratory failure requiring intubation. The patients were treated with subcutaneous injections of 300 micrograms of filgrastim or corresponding placebo (1 ml of 0.9% NaCl –solution) once daily for 7 days or until discharge from the ICU. If the neutrophil count exceeded 50x10^9^/L filgrastim/placebo was administered every other day and if neutrophil count exceeded 75x10^9^/L filgrastim/placebo was discontinued. The study personnel were blinded to study patients’ leukocyte and neutrophil counts until the data had been analyzed. Other intensive care treatments were performed according to the written standard operating procedures of the ICU.

Blood samples were drawn at study entry (baseline), day 4 and day 7 afterwards. The samples were taken before administration of the study drug. The sampling was performed only during the ICU stay and if a patient was discharged by day 7, blood collection was also discontinued. The blood samples were drawn and placed immediately in ice and centrifuged at +4°C. The plasma was stored in −70°C until further analysis. Total leukocyte count and absolute neutrophil count were determined in routine analysis of Helsinki University Central Hospital laboratory. The plasma HBP was determined by enzyme-linked immunosorbent assay as described earlier [2]. The detection limit of the method was 0.25 ng/ml and CV variance was <5%.

The primary endpoint of the main study were number of adverse events, the number of patients developing ARDS, disseminated intravascular coagulation or acute renal failure during days 1–28, and changes in MOD score [13]. For this substudy, the clinical data comprised of APACHE II [14], SOFA -score [15,16], admission diagnosis, and laboratory values for white blood cell (WBC), neutrophil count and inflammatory mediators: (Interleukin (IL)-6, Interleukin (IL)-10, soluble E-selectin (sE-selectin) and soluble Interleukin 2 receptor (sIL-2R) from baseline and day 3 [17].

### Statistical analysis

The data are presented as median and interquartile ranges (IQR) or absolute numbers and ranges, as appropriate. The normality of distribution of parameters was tested with Kolmorogov-Smirnov one-sample test. In case of non-normal distribution, the parameters were transformed to natural logarithm before analysis. The difference between groups is calculated by one-way analysis of variance with a p value <0.05 considered statistically significant. The correlations between parameters were calculated from values transformed to natural logarithm. Intraclass correlation coefficient (ICC) was used to examine the correlation between HBP -levels, P/F –ratio, SOFA –score, WBC and neutrophil count as well as inflammatory mediators.

## Results

Altogether 59 patients were included into the study, 30 in the filgrastim group and 29 in the placebo group. Due to an error in the preparation of randomisation envelopes number 45 was omitted and only 59 patients were randomised instead of the intended 60 patients. The baseline characteristics of the patients are presented in the Table 1.

**Table 1:** Baseline characteristics of study population

**Variable**	**Controls (n = 29)**	**Filgrastim (n = 30)**
Age (yr)	52 (28–73)	45 (20–76)
Gender (F/M)	6/23	9/21
APACHE II	13 (3–28)	11 (2–19)
Admission diagnosis		
Bacterial infection*	14	12
Inflammatory process**	6	6
Other	9	12
SOFA	9 (3–15)	8.5 (2–15)

APACHE, Acute Physiology and Chronic Health Evaluation; SOFA, Sequental Organ Failure Assessment, median (range).

*Bacterial infection: pneumonia, blood culture positive sepsis, meningitis, peritonitis.

**Inflammatory process: aspiration pneumonia, acute pancreatitis.

**Figure 1:**
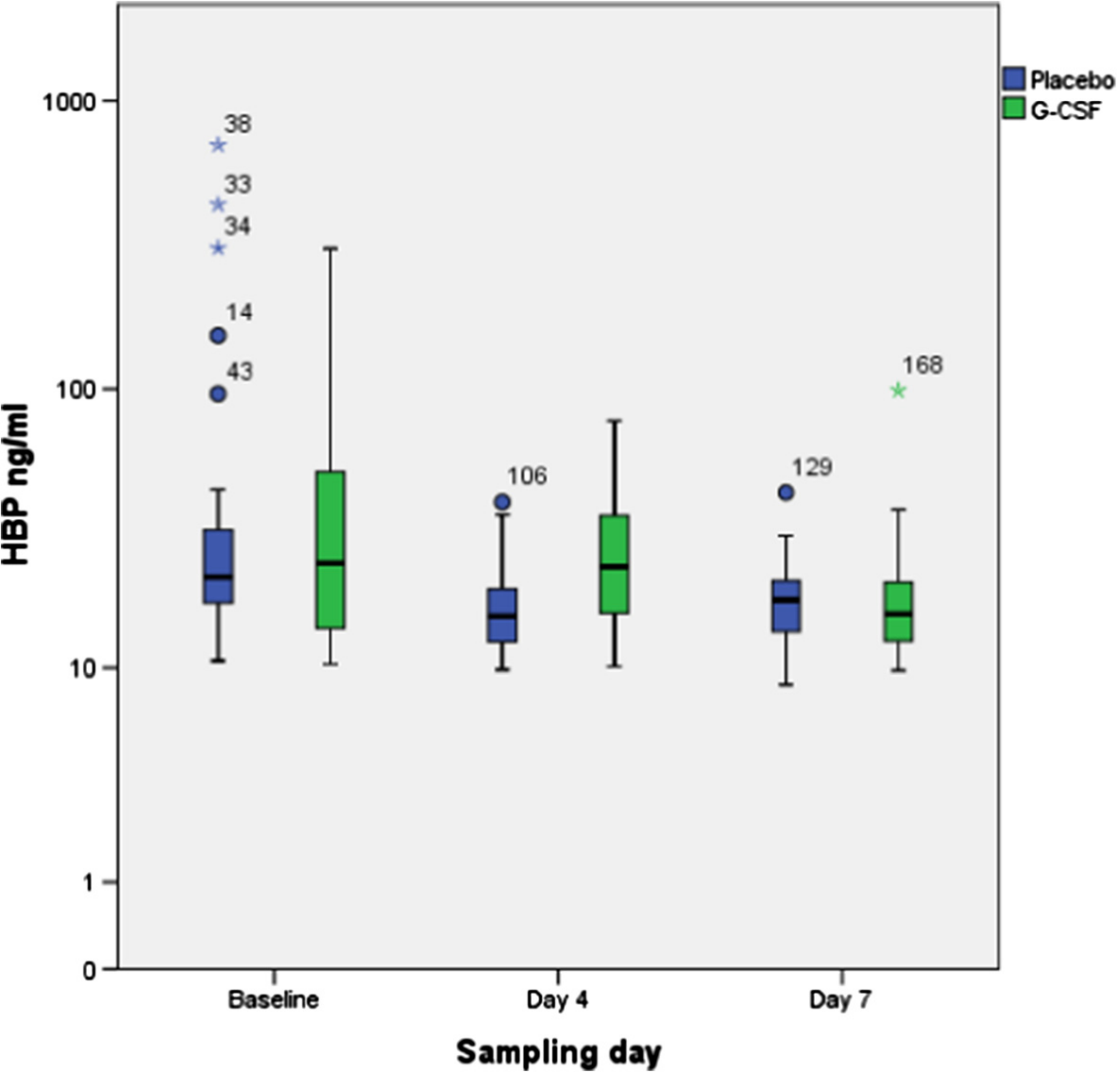
The concentrations (ng/ml; median, 95% CI) of HBP between patients receiving filgrastim 300 microgr/day or placebo for 7 days.

The plasma HBP –concentrations, absolute neutrophil counts and white blood cell counts at baseline, day 4 and day 7 are presented in Figure 1 and Table 2. The plasma concentrations of HBP in filgrastim and placebo groups were similar at baseline, median [IQR] 23.6 ng/ml [13.9-43.0 ng/ml] vs. 21.6 ng/ml [16.9-28.7 ng/ml], respectively (p = 0.785). At day 4, the filgrastim group had significantly higher HBP –concentrations when compared to placebo group 25.1 ng/ml [17.7-35.5 ng/ml] vs. 13.9 ng/ml [12.0-19.5 ng/ml], respectively (p < 0.05). At day 7, there was no difference in HBP levels 15.9 ng/ml [12.6-20.7 ng/ml] vs. 17.8 ng/ml [13.6-20.9 ng/ml] in filgrastim and placebo groups (p = 0.774), respectively.

**Table 2:** Concentrations of HBP, absolute neutrophil count and WBC in patients receiving daily filgrastim 300 microgr/day or placebo

	**Baseline**		**Day 4‡**		**Day 7**	
	**Filgrastim**	**Placebo**	**Filgrastim**	**Placebo**	**Filgrastim**	**Placebo**
HBP (ng/ml)	23.6 (13.9-43.0)	21.6 (16.9-28.7)	25.1 (17.7-35.5)*	13.9 (12.0-19.5)	15.9 (12.6-20.7)	17.8 (13.6-20.9)
Neutrophil count (x10^9^)/L	7.3 (4.0-11.1)	8.2 (3.6-12.8)	24.0 (14.6-32.8)†	9.4 (7.5-13.7)	27.1 (18.7-38.9)†	9.4 (6.1-12.0)
WBC count (x10^9^)/L	9.1 (5.6-12.3)	12.1 (4.3-17.1)	27.2 (17.6-36.0)†	12.1 (10.0-16.5)	29.7 (20.1-39.8)†	12.2 (14.9)
HBP/WBC –ratio	2.9 (1.4-8.7)	2.8 (1.5-11.0)	0.9 (0.6-1.7)*	0.7 (0.5-0.9)	1.6 (1.1-2.1) †	0.7 (0.7-1.0)
HBP/Neutrophil –ratio	4.0 (1.7-9.8)	3.0 (1.6-12.5)	1.2 (0.7-2.2)	1.8 (1.3-2.4)	0.7 (0.5-1.2)*	1.7 (1.3-2.1)

Median (IQR), WBC white blood cell count.

* p < 0.05 filgrastim vs. placebo.

† p < 0.001 filgrastim vs. placebo.

‡ Neutrophil count and WBC count from day 3.

The absolute neutrophil count and white blood cell counts were similar in filgrastim and placebo groups at baseline but at days 4 and 7 there were significant increase in filgrastim group (p < 0.01 for both; Table 2). The HBP/WBC –ratio was similar in both groups at baseline, but at days 4 and 7, the ratio decreased more in placebo group compared to filgrastim group. HBP/absolute neutrophil count –ratio also decreased from baseline in both groups, but the decrease was greater in filgrastim group (Table 2).

**Figure 2:**
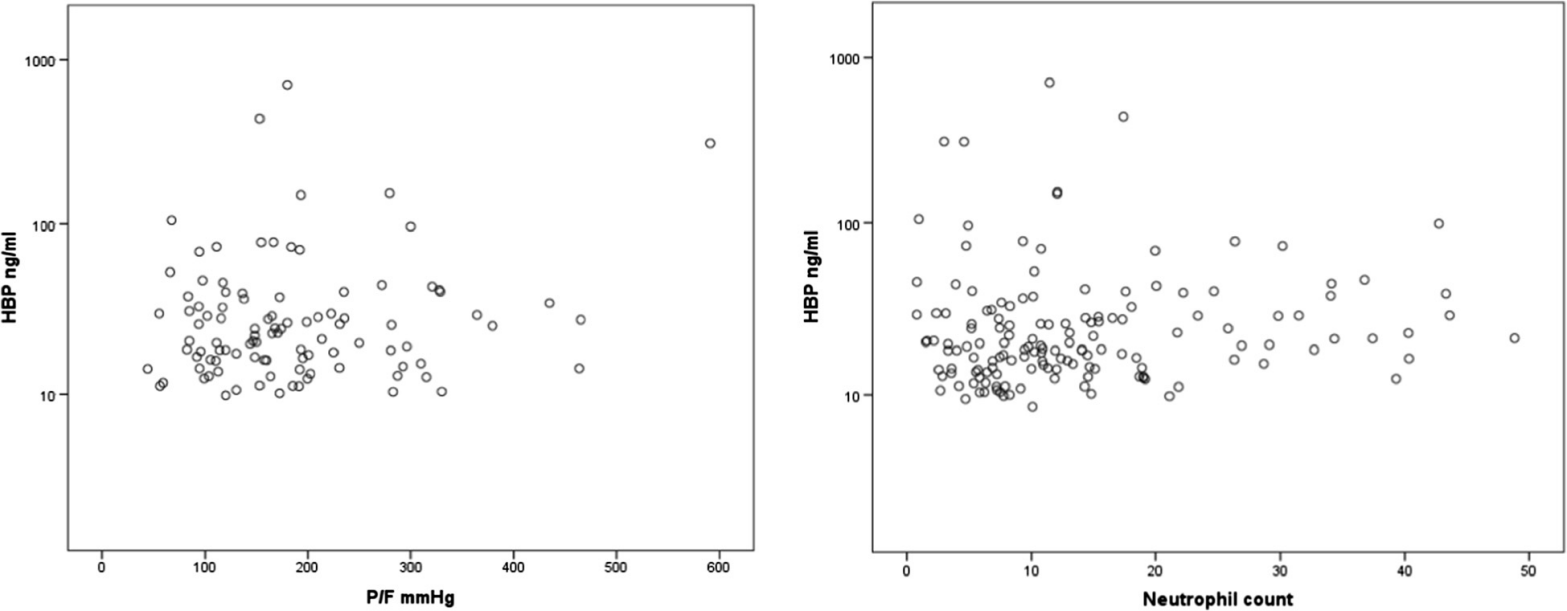
The correlation of HBP –concentrations to P/F –ratio (left panel) and absolute neutrophil count (right panel).

In patients with septic shock vs. no septic shock, no difference in plasma HBP –concentrations could be detected in any of the time points (baseline, 4 –day or 7 –day) regardless of filgrastim treatment. The HBP –concentrations were above 15 ng/ml in 44 of the 59 patients at baseline. No correlations between HBP –concentrations and absolute neutrophil count or P/F –ratio could be demonstrated (Figure 2). The concentrations of HBP (baseline or day 4) did not correlate with SOFA –score. No correlation of HBP with inflammatory mediators IL-6 (p = 0.42), IL-10 (p = 0.44), or sIL-2R (p = 0.32) was found. A modest correlation with sE-Sel was found (rho 0.275, p < 0.01).

## Discussion

Our study was the first to assess HBP –levels in critically ill patients receiving filgrastim or placebo in a controlled study. The HBP –levels were similar in both treatment groups at baseline, but differed at day 4. The difference in HBP –levels did not persist to day 7, and HBP –concentrations did not correlate with absolute neutrophil count or P/F –ratios.

The concentrations of HBP were elevated in both groups already at the baseline samples. In healthy volunteers without infections and in patients with local infections only, the concentrations of HBP have been around 7 ng/ml compared to 24 ng/ml and 22 ng/ml in filgrastim and placebo groups at baseline, respectively [8]. These baseline concentrations were, however, lower than those previously described for febrile patients at hospital admission who later develop septic shock (42 ng/ml) or severe sepsis (30 ng/ml) [5]. When compared to burn patients or sepsis patients at ICU admission, the HBP –levels were similar [4,7]. In our study population, all patients had acute respiratory failure, but not all had bacterial infection. HBP is released in systemic inflammatory reactions including sepsis, and a cut-off value of 15 ng/ml is found to have the best predictive value for the development of septic shock [5]. In our study patients 44 out of 59 exceeded this level at baseline even though only 16 of the 59 patients were diagnosed as having severe sepsis or septic shock.

HBP is an important mediator of bacterial infections transforming to septic shock. In septic situations, HBP is released from activated neutrophils by exocytosis of granule compartments. Filgrastim stimulates the production of new neutrophils in bone marrow and enhances the effector functions of mature neutrophils [18]. As HBP is released from activated neutrophils, the stimulation of neutrophil production and function by filgrastim could lead to increased secretion of HBP. In our study, the levels of HBP were already elevated at baseline compared to healthy volunteers or to patients with local infections only, but not compared to febrile patients who develop septic shock or severe sepsis. At day 4, there was a significant difference in the HBP –levels between filgrastim and placebo groups. This seems to be caused by a decrease of HBP –concentrations in placebo group rather than an increase in HBP –concentrations of filgrastim group. Previously, in burn patients, the levels of HBP have decreased to near-normal levels in 24–48 h after the ICU admission [4]. In our patients the high HBP –concentrations did not persist to day 7, at that day both groups had median HBP –levels lower than that at baseline.

The correlation of HBP concentrations to WBC count has been studied in previous trials [4,5]. In a subset of patients with severe sepsis (with or without septic shock), a positive correlation was found whereas no correlation was found in burn patients. In our patients, no correlations between WBC and HBP –levels was found. In previous trials the correlation between HBP –levels and absolute neutrophil count has not been studied. In our patient population, however, no such correlation could be verified.

The M1 –protein mediated release of neutrophil granule contents is responsible for subsequent lung injury [19]. As HBP is situated in secretory vesicles along with azurophilic/primary granule subsets, it is readily released when neutrophils become activated [2,10]. Initially filgrastim was thought to have beneficial effects on the outcome of critically ill patients with septic infections. However, no mortality benefit could be shown in critically ill patients with pneumonia and severe sepsis or septic shock in a large randomised placebo-controlled trial [20]. The worsening of pulmonary function and even the development of acute respiratory distress syndrome (ARDS) has been of concern in patients receiving filgrastim. As filgrastim treatment results in prolonged high levels of HBP, this could have deleterious effects on pulmonary capillary leakage and pulmonary function. In our patients, the plasma concentrations of HBP did not correlate with the P/F –ratios.

Our study had some limitations. The number of patients was low and, accordingly, there is considerable risk to type II error, i.e. not detecting a true difference even though it is present. Due to the limited number of patients in this randomized pilot study, we were not able to analyze HBP –levels in subgroups of patients with bacterial infections, other inflammatory processes, or with non-infectious causes of acute respiratory failure. Second, the sampling was not primarily designed for measurements of plasma HBP –concentrations, but rather to show the change in organ failure score before and after the intervention. As the decline in HBP occurs within days in critically ill patients [4], more frequent sampling would have been better to detect the maximal effect of filgrastim. Thus, it is possible that filgrastim may have caused a temporary increase in HBP –levels, and we were not able to detect it. Finally, as the primary study was performed already in 1997, the blood samples have been conserved in −70° over time. This may have had effect on the HBP levels. If degradation of HBP had occurred, it can be suspected that the degradation of HBP would have occurred to the same degree in both treatment groups.

## Conclusions

Filgrastim treatment is associated with increased circulating HBP levels compared to placebo, but the absolute neutrophil count or the degree of oxygenation failure did not correlate with the observed plasma HBP –concentrations.

### Key messages

Plasma levels of heparin-binding protein (HBP) were elevated in critically ill patients with acute respiratory failure requiring intubation at baseline.

A significant decrease in HBP –levels in placebo –treated patients was observed at day 4.

HBP –levels did not correlate with absolute white blood cell or neutrophil counts.

HBP –levels did not correlate with worsening of oxygenation measured by P/F –ratio.

## Abbreviations

HBP: Heparin-binding protein; IQR: Interquartile range; G-CSF: Granulocyte colony stimulating factor; ICU: Intensive care unit; APACHE II: Acute physiology and chronic health evaluation; SOFA: Sequential organ failure assessment; CV: Coefficient of variation; ARDS: Acute respiratory distress syndrome; DIC: Disseminated intravascular coagulopathy; WBC: White blood cell; ICC: Intraclass correlation coefficient; P/F –ratio: Arterial oxygen tension to fraction of inspired oxygen –ratio.

## Competing interests

Hansa Medical AB has filed a patent application on the use of HBP as a diagnostic tool in sepsis. HH is listed as inventor. KMK, LL and VP declare no competing interests.

## Authors’ contributions

KMK has performed statistical analysis of the HBP data and is responsible for the draft writing and submission of the manuscript. HH and LL have contributed in the planning of the study, are responsible for the determination of HBP –concentrations, contributed on the analysis of the data and writing of the manuscript. VP is responsible for the planning, execution and reporting of the original study and contributed in HBP data analysis and manuscript preparation. All authors have read and approved the final version of the manuscript.

## Pre-publication history

The pre-publication history for this paper can be accessed here:

http://www.biomedcentral.com/1471-2334/13/51/prepub
